# Cytoplasmic SET induces tau hyperphosphorylation through a decrease of methylated phosphatase 2A

**DOI:** 10.1186/1471-2202-15-82

**Published:** 2014-06-30

**Authors:** Stéphanie Chasseigneaux, Christine Clamagirand, Léa Huguet, Lucie Gorisse-Hussonnois, Christiane Rose, Bernadette Allinquant

**Affiliations:** 1 INSERM UMR 894, Université Paris Descartes, Sorbonne Paris Cité, Faculté de Médecine, Paris, France; 2 Inserm, U1144, Paris F-75006, France; 3 Université Paris Descartes, UMR-S 1144, Paris F-75006, France; 4 Université Paris Diderot, UMR-S 1144, Paris F-75013, France

**Keywords:** Cytoplasmic SET, Protein Phosphatase 2A, Methyl Phosphatase 2A, Tau hyperphosphorylation, Alzheimer disease

## Abstract

**Background:**

The neuronal cytoplasmic localization of SET, an inhibitor of the phosphatase 2A (PP2A), results in tau hyperphosphorylation in the brains of Alzheimer patients through mechanisms that are still not well defined.

**Results:**

We used primary neurons and mouse brain slices to show that SET is translocated to the cytoplasm in a manner independent of both its cleavage and over-expression. The localization of SET in the cytoplasm, either by the translocation of endogenous SET or by internalization of the recombinant full-length SET protein, induced tau hyperphosphorylation. Cytoplasmic recombinant full-length SET in mouse brain slices induced a decrease of PP2A activity through a decrease of methylated PP2A levels. The levels of methylated PP2A were negatively correlated with tau hyperphosphorylation at Ser-202 but not with the abnormal phosphorylation of tau at Ser-422.

**Conclusions:**

The presence of full-length SET in the neuronal cytoplasm is sufficient to impair PP2A methylation and activity, leading to tau hyperphosphorylation. In addition, our data suggest that tau hyperphosphorylation is regulated by different mechanisms at distinct sites. The translocation of SET to the neuronal cytoplasm, the low activity of PP2A, and tau hyperphosphorylation are associated in the brains of Alzheimer patients. Our data show a link between the translocation of SET in the cytoplasm and the decrease of methylated PP2A levels leading to a decrease of PP2A activity and tau hyperphosphorylation. This chain of events may contribute to the pathogenesis of Alzheimer disease.

## Background

Hyperphosphorylation of tau, which leads to the formation of neurofibrillary tangles, is one of the two hallmarks of Alzheimer disease (AD) [1-3]. Tau phosphorylation is mainly regulated by the balance of kinases/phosphatases. Several kinases are involved in tau phosphorylation including the glycogen synthase kinase 3β (GSK-3β), MAP kinase (MEK1/2), neuronal cdc2-like kinase (NCLK) and cyclin dependent kinase 5 (cdk5) [1-3]. In contrast, few phosphatases dephosphorylate tau. Phosphatase 2A (PP2A), a serine/threonine phosphatase, is the most effective.

PP2A is a trimeric protein with a heterodimeric core enzyme consisting of a catalytic C subunit (PP2Ac), and a scaffolding A subunit. This core enzyme associates with a variable regulatory subunit to form a heterotrimeric holoenzyme [4-6]. PP2A shows efficient enzymatic activity when it is methylated at Leu-309 [7-10] and unphosphorylated at Tyr-307 [11]. In the brain of AD patients, the activity of PP2A is compromised [12-14]. The decrease of PP2A methylation observed in brains of AD patients [15] may explain the decrease of PP2A activity and contribute to tau hyperphosphorylation [16,17].

The activity of PP2A can be inhibited by two proteins: I1PP2A and I2PP2A, which is also called SET, TAF1β, or PHAPII [18]. SET is a multifunctional protein: it inhibits the acetylation of histones [19], blocks DNase activity [20], acts as a transcription factor [21], and promotes tau phosphorylation by activating cdk5 [22], and inhibiting PP2A [23].

In the brains of AD patients, there is an increase of SET expression [24,25]. Interestingly, SET is also translocated from the nucleus to the cytoplasm in the hippocampus and the temporal cortex of AD patients [25,26]. We observed that in the CA1 of AD patients, the translocation of SET to the cytoplasm was associated with an increase of Amyloid Precursor Protein (APP) cleaved in its cytoplasmic domain by a caspase (APPcc) [26]. In *in vitro* models, cytoplasmic SET is associated with neuronal death [27-29] and with tau hyperphosphorylation [30,31]. The 39 kDa full-length SET can be selectively cleaved resulting in a ~ 20 kDa fragment in the cytosol of neurons in the brain [25]. The cleavage of SET protein has also been observed in primary neurons treated with kainate and in a mouse model of stroke [32]. This cleavage results from the activation of an asparaginyl endopeptidase (AEP) which cuts SET at asparagine Asn-175, generating NTF and CTF fragments and triggering DNA nicking and cell death [33]. Both NTF and CTF are able to bind to the catalytic subunit of PP2A (PP2Ac) inhibiting its activity and leading to tau hyperphosphorylation [34-36]. However, it is not clear whether the cytoplasmic localization of SET is always associated with its cleavage, with its over-expression, and with tau hyperphosphorylation.

It is still not clear how cytoplasmic SET contributes to PP2A loss of function leading to tau hyperphosphorylation, and whether the presence of SET in the cytoplasm induces low levels of methylated PP2A. We used two models to clarify the relationship between cytoplasmic SET, methylated PP2A, PP2A activity and tau hyperphosphorylation. The first model involved the translocation of endogenous SET from the nucleus to the cytoplasm in primary neurons or brain slices from wild type mice (WT). This translocation was induced in this model by the internalization of the Jcasp peptide. Indeed, this peptide mimics the unmasked juxtamembrane cytoplasmic domain arising from the cleavage of APP by caspases, which is increased in the brains of AD patients [37-39,26]. Moreover, this peptide is sufficient to induce *in vivo* both translocation of endogenous SET, as occurs in the CA1 of WT mice following APPcc overexpression, and neurodegeneration [26,27,40]. The second model involved the over-expression of SET by the internalization of exogenous recombinant full-length protein in brain slices from WT mice [27]. In these two models, we report that cytoplasmic SET induces the hyperphosphorylation of tau in the absence of detectable cleaved forms of SET. We also show that the interaction of SET with PP2A impairs the methylation of PP2A and that the level of methylated PP2A that is associated with the translocation of SET is also negatively correlated with the hyperphosphorylation of tau at Ser-202, but not at Ser-422, suggesting that the hyperphosphorylation of tau is regulated by different mechanisms at distinct residues.

## Results

### Internalization of Jcasp peptide induces the translocation of endogenous nuclear SET to the cytoplasm without cleavage or upregulation of its expression

We previously reported that the cytoplasmic internalization of the Jcasp peptide by primary neurons resulted in the translocation of endogenous SET to the cytoplasm and triggered pro-apoptotic signals at the cell membrane [27,28]. Thus, in this model, SET promotes apoptosis, and its translocation to the cytoplasm appears to participate in the active neurodegenerative process [27,28].

**Figure 1:**
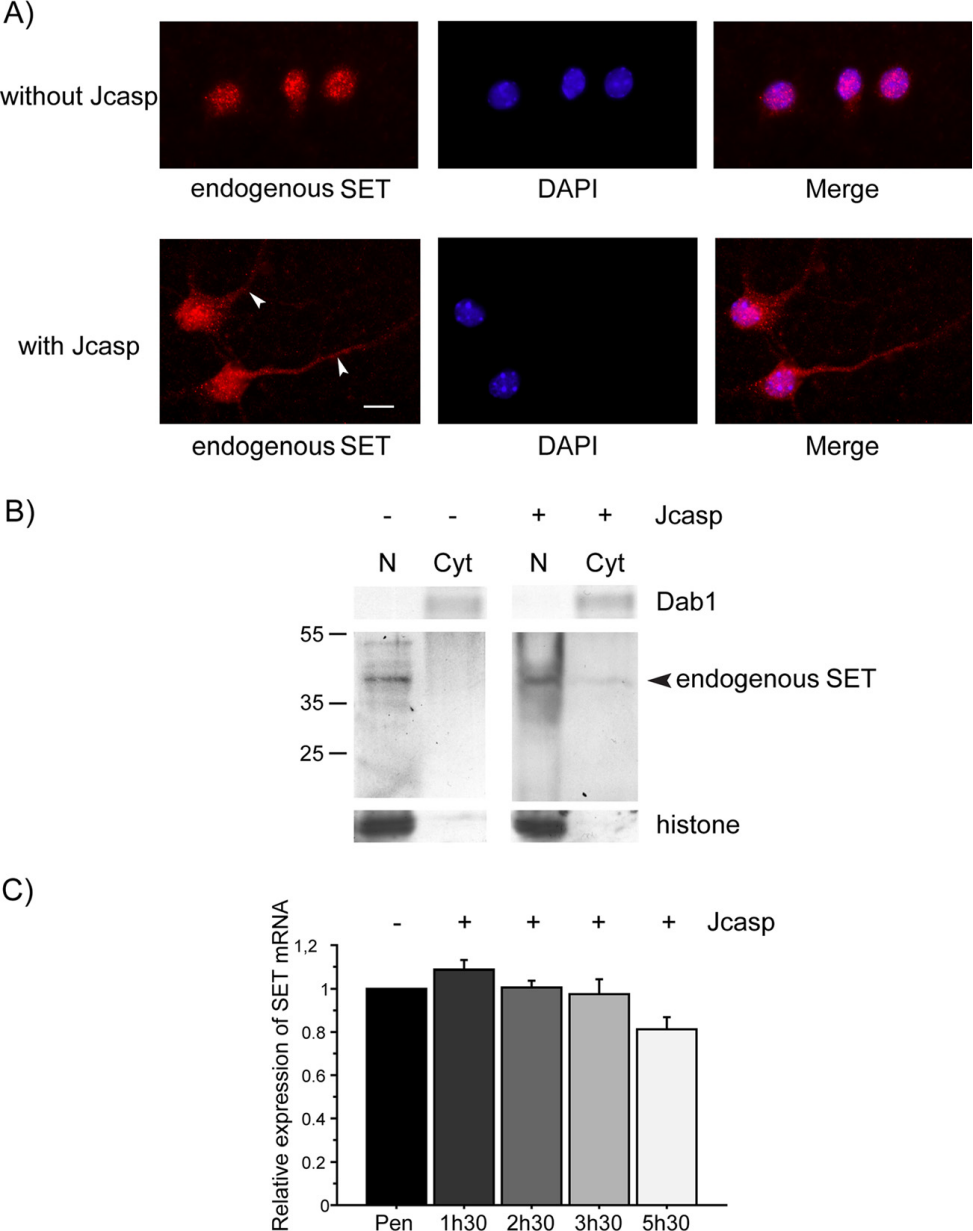
**Endogenous SET translocation after 5 h 30 min of Jcasp peptide internalization in mouse primary neurons. A)** Immunocytochemical staining (epifluorescence) of endogenous SET (Cy3) in primary neurons treated with the Jcasp peptide (2 μM) for 5 h 30 min, and control neurons in the absence of any peptide. Nuclei were labeled with DAPI. SET is mostly present in the nucleus of control neurons in the absence of any peptide as indicated in combined images (Merge), whereas SET translocation to the cytoplasm occurs after the internalization of Jcasp peptide (see arrowheads). One representative immunocytochemical staining out of five independent experiments is presented. Scale bar, 10 μm. **B)** Representative western blot of cytoplasmic (Cyt) and nuclear (N) neuronal extracts obtained by sub-cellular fractionation (n = 3 independent experiments). Endogenous SET was detected with a SET antibody. Dab1 was used as a cytoplasmic control and histone was used as a nuclear control. A total of 10 μg of protein was loaded in each lane. Endogenous SET at 39 kDa is partially localized in the cytoplasm in cells containing Jcasp peptide. Cleaved SET fragments cannot be detected. **C)** Relative expression of SET mRNA assessed by quantitative real-time PCR in cells treated with Jcasp peptide for 1 h 30 min, 2 h 30 min, 3 h 30 min and 5 h 30 min and with the Penetratin (Pen) peptide only at the same time points. Results are expressed as the ratio of SET mRNA in Jcasp peptide internalization versus Penetratin only assays at the different time points. Data presented are the mean ± SEM of three independent experiments. Jcasp peptide does not affect the level of SET mRNA.

In this model, we also previously showed that the Jcasp peptide fused to the Penetratin vector is quickly internalized, and that endogenous SET starts to move from the nucleus to the cytoplasm after 3 h, while apoptosis was observed 24 h later [27]. Here, we show that translocated SET is still present in the cytoplasm of the cellular body and in neurites 5 h 30 min after Jcasp peptide internalization (Figure 1A). Sub-cellular fractionation and western blotting revealed the presence of a 39 kDa band corresponding to endogenous SET in the cytoplasm fraction of cells treated with Jcasp peptide (Figure 1B). The proportion of translocated SET was 39% of total endogenous SET (Figure 1B), which was in accordance with observations made by epifluorescence microscopy (Figure 1A). Cleavage products of SET in the nucleus and in the cytoplasm were not detected (Figure 1B). Real-time PCR showed that the level of SET transcripts was unchanged during the 5 h 30 min of Jcasp peptide internalization (Figure 1C).

Hence, in this model, the translocation of SET from the nucleus to the cytoplasm does not require its over-expression and/or its cleavage.

### Over-expression of recombinant cytoplasmic SET in primary mouse neurons and mouse brain slices does not induce its cleavage

**Figure 2:**
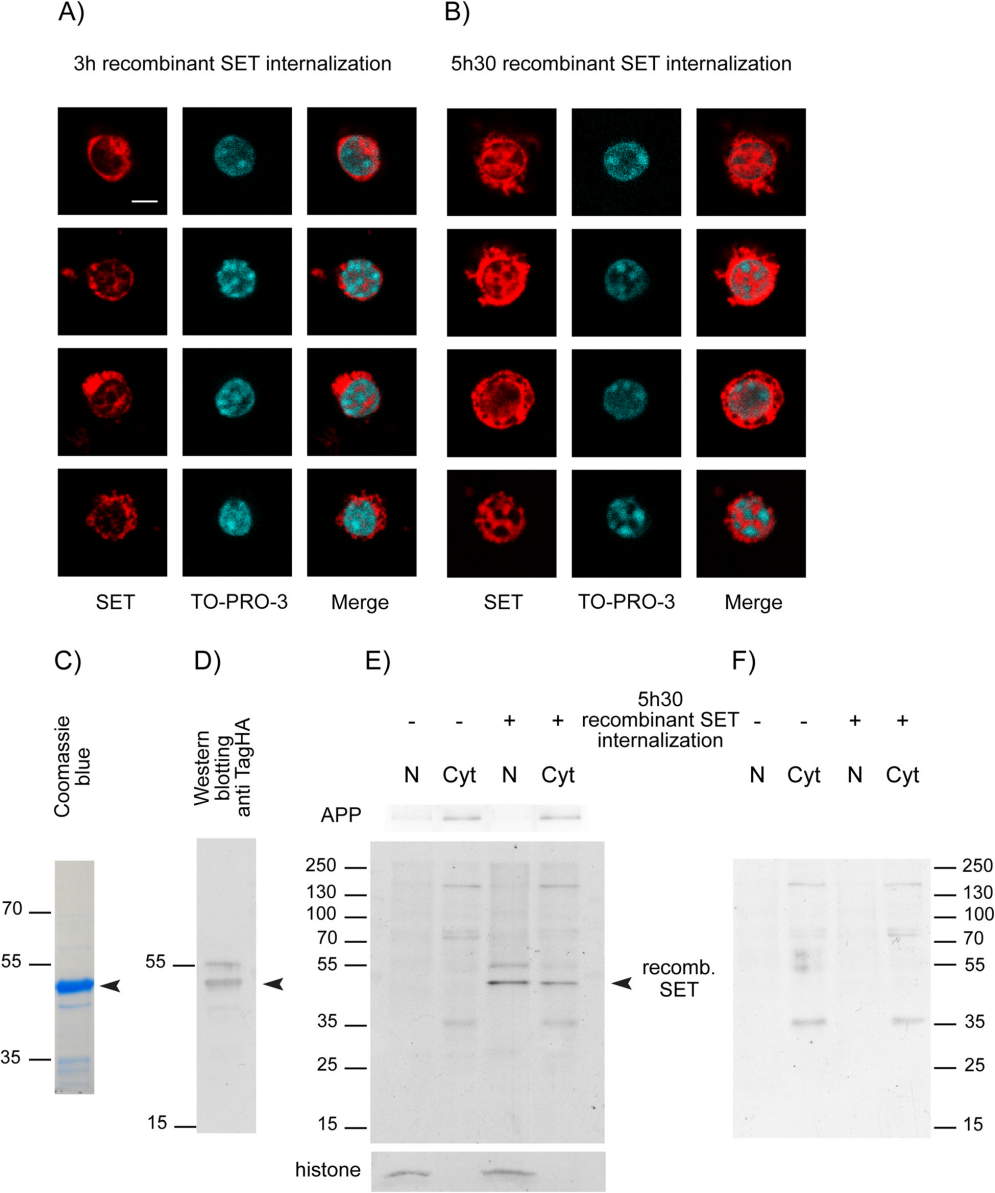
**Localization of the recombinant SET protein after its internalization.** Recombinant SET protein (4 μM) was internalized for 3 h and 5 h 30 min in mouse primary neurons **(A, B)** and for 5 h 30 min in mouse brain slices **(E, F)**. Confocal microscopy shows immunocytochemical staining of recombinant SET carried-out with an anti-tag HA antibody. Nuclei were labeled with TO-PRO-3. Four examples at 3 h **(A)** and at 5 h 30 min **(B)** are shown. At 3 h, recombinant SET protein is mostly located in the cytoplasm around the nucleus, whereas at 5 h 30 min SET is distributed in both the cytoplasm and nucleus. One representative immunocytochemical staining out of three independent experiments is presented. Scale bar, 10 μm. **(C)** Electrophoresis of tag-HA recombinant SET protein (10 μg of protein was loaded). Coomassie blue shows a major band at 49 kDa, as expected. **D-F)** Tag-HA recombinant SET protein (0.4 μg) was detected by western blotting with a tag-HA antibody **(D)**. Sub-cellular fractions were prepared from brain slices treated for 5 h 30 min with recombinant SET protein and 10 μg of proteins were loaded in each lane. Recombinant SET was detected with a tag-HA antibody **(E)**. APP was used as a cytoplasmic (Cyt) control and histone was used as a nuclear (N) control. Recombinant full-length SET protein is observed at 49 kDa in nuclear and cytoplasmic fractions as in **(B)**. Unspecific bands recognized by the secondary antibody are shown in **(F)**. For **(D-F)**, one representative immunoblot out of five independent experiments is shown and for **(C)**, one protein staining of recombinant SET out of three independent experiments is presented **(C)**.

We introduced a recombinant HA-tagged SET protein fused to the Penetratin vector into the cytoplasm of primary neurons by direct internalization as previously described [27]. We detected the 49 kDa recombinant SET protein by immunocytochemistry with an anti-HA antibody and analyzed its distribution by confocal microscopy. After 3 h of internalization, the recombinant SET protein, which possesses a nuclear targeting sequence, was mostly concentrated in the cell body around the nucleus and had started to move inside the nucleus (Figure 2A). After 5 h 30 min of internalization, the nucleus was enriched with the recombinant SET protein, although recombinant SET was still abundantly present in the cytoplasm (Figure 2B). We confirmed these observations with mouse brain slices that were treated with the recombinant SET protein. Sub-cellular fractionation followed by western blotting with an anti-HA antibody revealed the presence of recombinant SET in the cytoplasm (Figure 2C-F). The detected bands of low molecular weight and low intensity were unspecific bands resulting from the anti-rabbit secondary antibody (Figure 2F). We did not observe cleaved recombinant SET protein in either sub-cellular compartment studied (Figure 2E).

### Interaction between uncleaved cytoplasmic recombinant SET protein and PP2A inhibits the activity of PP2A and impairs its methylation in the brain slices of adult mice

**Figure 3:**
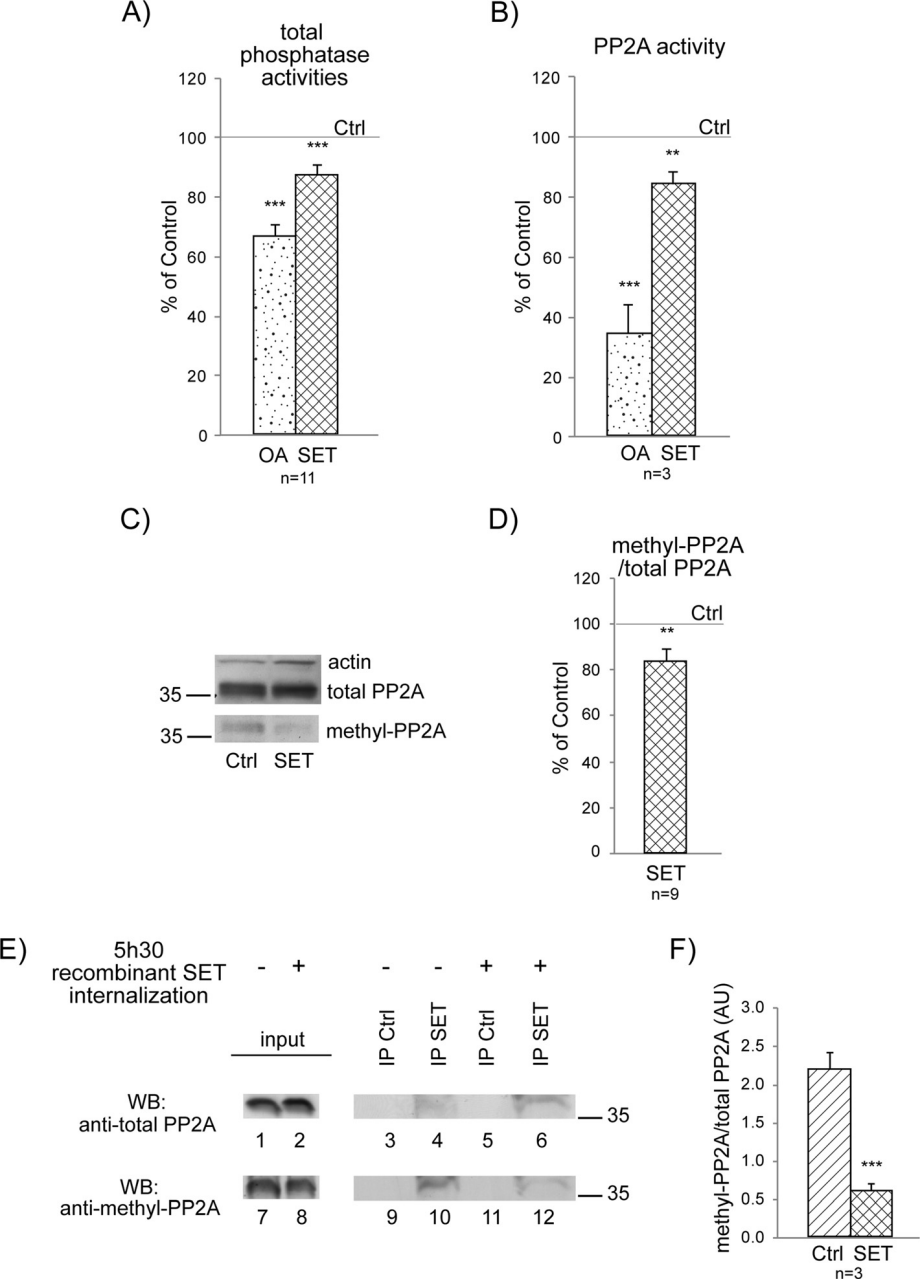
**Phosphatase activities and PP2A methylation in adult mouse brain slices treated with recombinant SET protein for 5 h 30 min. A)** The release of p-nitrophenol (nmoles of p-nitrophenol/mg of protein) was assessed. Results are expressed as a percentage of control (Ctrl) activity that was set to 100%. Mean ± SEM of 11 independent experiments; okadaic acid (OA); OA *versus* Ctrl: *p* < 0.0001; SET *versus* Ctrl: *p* = 0.0008. **B)** Release of p-nitrophenol measured on immunoprecipitated PP2A. Mean ± SEM of three independent experiments; OA *versus* Ctrl: *p* = 0.0025; SET *versus* Ctrl: *p* = 0.0124. **C)** Representative western blot (40 μg of protein loaded) of total and methylated PP2A (methyl-PP2A) and quantification of the ratio of methylated PP2A to total PP2A **D)**. Mean ± SEM of nine independent experiments. SET *versus* Ctrl: *p* = 0.0057. **E)** Representative western blot of total PP2A and methyl-PP2A co-immunoprecipitated with a SET primary antibody (out of three independent experiments). Mouse brain slices in the absence (-) or the presence of recombinant SET protein (+) were immunoprecipitated with a SET antibody. Co-immunoprecipitated proteins were analyzed by western blotting, with a total PP2A antibody (lanes 3–6) and with a methylated PP2A (methyl-PP2A) antibody after membrane stripping (lanes 9–12). PP2A (lanes 3 and 5) and methyl-PP2A (lanes 9 and 11) are not detectable in absence of primary SET antibody (IP Ctrl). Similar levels of total PP2A (lanes 1 and 2) and methyl-PP2A (lanes 7 and 8) in total extracts before SET immunoprecipitation. **F)** Quantification of the ratio of methyl-PP2A to total PP2A co-immunoprecipitated with SET primary antibody in mouse brain slices treated with recombinant SET protein for 5 h 30 min and control (Ctrl) brain slices (no internalization). Meam ± SEM of three independent experiments; SET *versus* Ctrl: *p* = 0.0027.

Following its translocation to the cytoplasm, endogenous SET interacts with the catalytic subunit of PP2A inducing its inhibition [23,34]. We used direct internalization for 5 h 30 min to overexpress recombinant SET protein (4 μM) in adult mouse brain slices maintained under oxygenation conditions, as described in Methods. We first analyzed total phosphatase activity (Figure 3A). We used okadaic acid (OA), a marine sponge toxin which is an inhibitor of PP2A, as a positive control (1 μM for 2 h) [41]. OA inhibited total phosphatase activity by 33.6% ± 3.7% (n = 11) and recombinant SET inhibited total phosphatase activity by 13.1% ± 3.3% (n = 11). Hence, phosphatase activity was significantly impaired in both cases and was 68.4% ± 3.7% of the baseline value (standardized to 100%) for OA (n = 11, *p* < 0.0001), and was 86.9% ± 3.3% for recombinant SET (n = 11, *p* = 0.0008) (Figure 3A). We then evaluated PP2A activity (which represents 70% of total phosphatase activity [42]) after PP2A immunoprecipitation in brain slices treated with OA and in those containing internalized recombinant SET protein. The remaining PP2A activity was 33.8% ± 9.8% (n = 3, *p* = 0.0025) in brain slices treated with OA and 84% ± 3.8% in brain slices containing recombinant SET (n = 3, *p* = 0.0124) (Figure 3B). These values fit with what we observed for total phosphatase activity. Thus, these data suggest that when SET is in the cytoplasm, it specifically impairs PP2A activity in a manner that does not depend on its cleavage (Figure 2E).

The activity of PP2A depends on its methylation [7-10]. Therefore, we assessed whether the interaction between cytoplasmic recombinant SET protein and endogenous PP2A impaired the methylation of PP2A, resulting in a decrease in its activity. We used an antibody directed against total PP2A (methylated and not methylated) and an antibody specifically directed against methyl-PP2A, and evaluated the ratio of methyl-PP2A to total PP2A by western blotting (Figure 3C, D). After 5 h 30 min of recombinant SET protein internalization, the ratio of methyl-PP2A to total PP2A was 83.6% ± 5.2% (n = 9) of the control ratio (standardized to 100%) in the absence of recombinant SET (Figure 3D). The mean decrease of this ratio (16.4%) was significant (*p* = 0.0057) suggesting that the presence of SET in the cytoplasm in the absence of detectable SET cleavage impairs the methylation of PP2A.

Finally, we performed co-immunoprecipitation experiments to check that the internalized recombinant SET protein interacted with PP2A and impaired the methylation of PP2A. To this end, we internalized the recombinant SET protein in brain slices for 5 h 30 min, and we extracted proteins from these slices and from control slices not internalized with recombinant SET. After lysis, we checked first the total level of PP2A (Figure 3E, lanes 1, 2) and methyl-PP2A (Figure 3E, lanes 7, 8) in brain slices by western blotting. We subsequently immunoprecipitated SET from slice extracts with an antibody that recognizes both endogenous and recombinant SET. We then analyzed the immunoprecipitates by western blotting with an antibody that recognizes total PP2A (Figure 3E, lanes 3–6) and after membrane stripping, with an antibody that specifically recognizes methyl-PP2A (Figure 3E, lanes 9–12). Only a small amount of PP2A co-immunoprecipitated with the SET antibody (Figure 3E, lane 4) in extracts from brain slices lacking recombinant SET, because only a small proportion of endogenous SET is cytoplasmic. More PP2A co-immunoprecipitated with SET in cells containing recombinant SET protein than in cells lacking the recombinant protein (Figure 3E, lane 6), showing that PP2A can bind to both recombinant and endogenous SET protein. In contrast, more methyl-PP2A co-immunoprecipitated with SET in the control (Figure 3E, lane 10) than in cells containing recombinant SET protein (Figure 3E, lane 12). The mean ratio of methyl-PP2A to total PP2A was 2.18 ± 0.22 (n = 3) for the control (Figure 3E, lane 10 to lane 4) and 0.59 ± 0.10 for brain slices treated for 5 h 30 min with recombinant SET protein (Figure 3E, lane 12 to lane 6). Thus, the level of co-immunoprecipitated methylated PP2A was significantly lower in slices internalized with SET protein than in the control (*p* = 0.0027) (Figure 3F). These data confirm that cytoplasmic, uncleaved, recombinant SET protein interacts with PP2A and impairs its methylation.

### Uncleaved SET in the cytoplasm induces hyper- and abnormal phosphorylation of tau protein. Correlation with decreased methyl-PP2A levels

Many phosphorylation sites of tau in paired helical filaments (PHF) have been identified in the brains of AD patients [1-3]. Inhibition of PP2A leads to the preferential hyper-phosphorylation of tau at various epitopes. Phosphorylation at Ser-202 and Ser-404 is more prevalent in PHF-tau and fetal tau than in normal adult tau [43,44]; therefore, we analyzed hyper-phosphorylation of tau at both these sites. We then focused on Ser-202 because PP2A alone regulates dephosphorylation at this site [44]. We used the AT8 antibody, which is commonly used for the immunohistological analysis of AD patient brains. This antibody can recognize monophosphorylated tau at Ser-202 [45]. We also focused our analysis on the abnormal phosphorylation at Ser-422, because the phosphorylation of this site is observed only in PHF-tau and is almost undetectable in biopsy-derived adult human tau [46].

**Figure 4:**
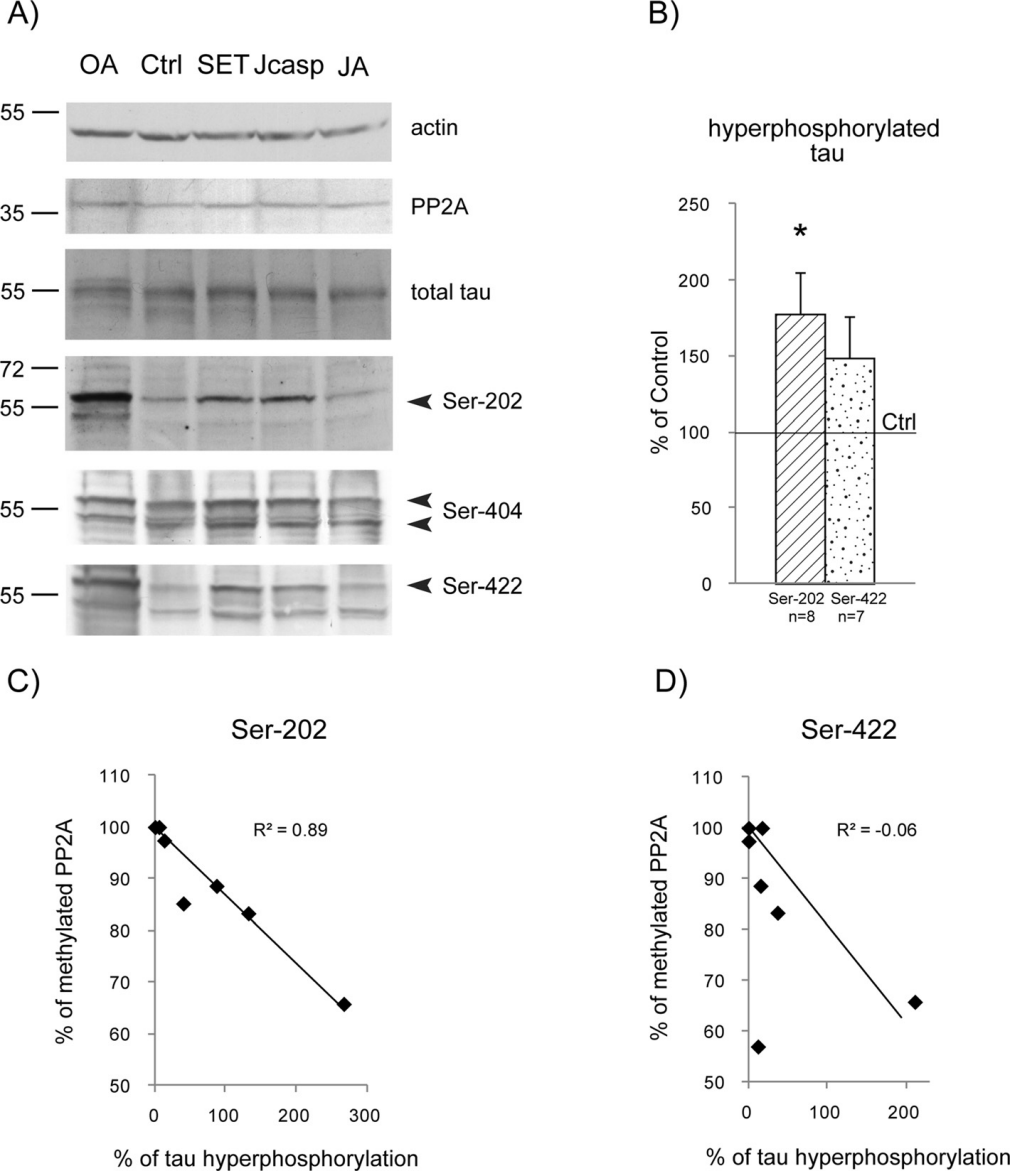
**Phosphorylation of tau in adult mouse brain slices treated with recombinant SET protein or Jcasp peptide for 5 h 30 min and its relationship with methylated PP2A. A)** Proteins were extracted from mouse adult brain slices treated with recombinant SET protein or Jcasp peptide and were analyzed by western blotting (40 μg per lane). Representative western blots of tau hyperphosphorylation at Ser-202, Ser-404 and Ser-422 and total tau protein are shown. Brain slices not treated with any protein (Ctrl) and brain slices treated with mutated Jcasp (JA) were used as negative controls. Okadaic acid (OA) was used as positive control. PP2A was also analyzed. Actin was used as internal loading control. Jcasp or SET internalization induces hyperphosphorylation of the same tau isoforms. **B)** Quantification of hyperphosphorylation of Ser-202 (n = 8 independent experiments) and Ser-422 (n = 7 independent experiments) in brain slices treated with SET relative to the phosphorylation of tau at these residues in control cells (set to 100%). Results were normalized to total tau. **C-D)** Correlation between tau hyper-phosphorylation at Ser-202 **(C)** (n = 8 independent experiments) and Ser-422 (n = 7 independent experiments) **(D)** and methylated PP2A. Phosphorylation at Ser-202 is significantly negatively correlated with methylated PP2A in cells containing recombinant SET.

We prepared lysates from brain slices internalized with Jcasp peptide or recombinant SET protein for 5 h 30 min and analyzed tau phosphorylation by western blotting with various antibodies (see Methods) directed against the epitopes mentioned above. We used OA, which increases tau phosphorylation at several physiological sites and induces phosphorylation at abnormal sites, as a positive control. We used the JA peptide as a negative control; this peptide is a mutated form of the Jcasp peptide in which a tyrosine is replaced with an alanine resulting in a peptide that induces neither the deleterious effect of the Jcasp peptide nor SET translocation [40,27]. We observed an increase of phosphorylated tau in cells containing the Jcasp peptide or recombinant SET protein (Figure 4A) whereas tau phosphorylation in cells containing the JA peptide was similar to that of the control. We defined tau phosphorylation of the control as 100%. Hyper-phosphorylation at Ser-202 that was induced by the recombinant SET protein was 177.6 ± 27.1% (n = 8; *p* = 0.02), and that induced by recombinant SET at Ser-422 was 148.3 ± 27.3% (n = 7) (Figure 4B). However the hyperphosphorylation at Ser-422 was lower than that at Ser-202, except for one animal out of the seven studied. The hyper- and/or abnormal phosphorylation of tau protein induced by recombinant SET protein or Jcasp peptide usually involve the same tau protein isoforms as those affected by OA. However, in some experiments, the isoforms affected by OA differed from those affected by recombinant SET protein or the Jcasp peptide, as observed for the 50 kDa tau isoform at Ser-404 (Additional file 1: Figure S1A), Ser-356, and Ser-235 (Additional file 1: Figure S1A) and the 70 kDa tau isoform at Ser-202 (Additional file 1: Figure S1B). The level of PP2A was similar in all conditions (Figure 4A). Interestingly, we found a significant negative correlation between the percentage of methylated PP2A and the percentage of tau protein hyper-phosphorylated at Ser-202 that was detected with the AT8 antibody (R^2^ = 0.89, n = 7) (Figure 4C). However, there was no correlation with the percentage of tau protein abnormally phosphorylated at Ser-422 (R^2^ = -0.06, n = 7) (Figure 4D), confirming that PP2A preferentially acts on the phosphorylation of tau at Ser-202.

## Discussion

We report here that the presence of SET in the cytoplasm, in absence of its over-expression or cleavage, decreases the activity of PP2A and thus stimulates tau hyperphosphorylation. Cytoplasmic SET impairs the methylation of PP2A, and the level of methylated PP2A is negatively correlated with tau hyperphosphorylation at Ser-202 but not at Ser-422.

An increase of SET expression leading to an increase of SET protein has been described in the brains of AD patients, and is associated with the cytoplasmic translocation of SET [25]. More recently, we also observed an increase of SET associated with its cytoplasmic translocation in the hippocampus of Down syndrome patients and in the CA1 of AD patients [26]. However, we cannot exclude that particular stress signals trigger the delocalization of SET in the absence of its over-expression in some subregions of the brains of AD patients [26]. The translocation of endogenous SET was induced by a cell death signal in the Jcasp model [28] in absence of SET over-expression. Similarly, we reported that the over-expression of APPcc with a lentiviral construct in the CA1 of mice resulted in the translocation of endogenous SET in the absence of SET increase [26]. Phosphorylation of SET at Ser-9 by casein kinase II causes the cytoplasmic retention of SET in the neuronal cytoplasm of the brains of AD patients [47]. We did not investigate the phosphorylation of SET at Ser-9 following the translocation of SET from the nucleus to the cytoplasm, or after its uptake by neurons.

The role of SET cleavage is not clear. Full-length SET and its cleaved forms can bind to PP2A, inhibiting its activity and thus increasing tau phosphorylation [34]. It is not clear whether this cleavage occurs in the nucleus or in the cytoplasm and whether both fragments remain in the same subcellular compartment. However, transfection experiments show that full-length SET is found within the nucleus as expected, whereas the NTF is distributed across cell compartments and the CTF is concentrated in the nucleus but is also present in the cytoplasm [34]. A recent report showed that the level of asparaginyl endopeptidase, which cleaves SET at Asn-175, is increased in the brains of AD patients [33]. Moreover, asparaginyl endopeptidase translocates from neuronal lysosomes to the cytoplasm where SET can be cleaved, an event that was observed under acidic conditions *in vitro* and *ex vivo*[33]. The absence of acidic conditions in the *ex vivo* models used in this report may explain why SET was not cleaved. We cannot exclude that SET is somewhat cleaved in *in vivo* conditions. Nonetheless, we show that cytoplasmic, full-length SET is sufficient to decrease PP2A activity and induce tau hyperphosphorylation, regardless of the route of entry of SET to the cytoplasm.

Caspases cleave APP at Asp-664 (APPcc). An increase of APPcc has already been shown in the brains of AD patients [37-39]. APPcc may be cleaved further into amyloid peptide resulting in the release of fragments containing the Jcasp sequence. APP intracellular domain generated after amyloid formation, can also be cleaved by caspases, leading to the formation of analogs of the Jcasp sequence. We found that the delivery of the Jcasp peptide to the cytoplasm was sufficient to induce the translocation of SET and the hyper-phosphorylation of tau, similar to what we observed with the over-expression of APPcc [26]. Recently, Asp-664 cleavage of APP in N2A cells and primary hippocampal neurons was shown to induce tau hyperphosphorylation by decreasing PP2A activity [48].

The activity of PP2A is low in the brains of AD patients [12-14]. Specific inhibitors of PP2A such as I1PP2A and SET, if localized in the cytoplasm, may underlie this impaired activity. The low activity of PP2A in the brains of AD patients may be also partly related to the low level of PP2A methylated at Leu-309 of the PP2Ac [15,17]. The methylation of this residue is also impaired in the cortex of transgenic APPxPS1 mice, in the brains of mice with mutated estrogen receptors, and after the treatment of N2A cells carrying the human APP Swedish mutation with OA [17]. We report here that full-length SET in the cytoplasm interacts with PP2A and decreases its methylation, thus impairing PP2A activity that leads to tau hyper-phosphorylation. Our co-immunoprecipitation experiments confirm the interaction of PP2A with full-length SET that was reported previously [34]. This interaction may impair the methylation of PP2A by inducing a conformational change that either prevents the interaction of PP2A with the leucine carboxyl methyltransferase 1 or stimulates its interaction with the PP2A specific methylesterase PME-1 [49-51]. Alternatively, the interaction of PP2A with SET may lead to a change of localization of PP2A, which may be preferentially redistributed to non-raft membrane domains where PP2A methylesterase PME-1 is exclusively present [51].

The physiological cellular state of phosphorylation results from an equilibrium between phosphatases and kinases. Dephosphorylation at Ser-202 and Ser-422 is dependent on PP2A [3]. Interestingly, we established a negative correlation between the level of methylated PP2A and that of tau phosphorylated at Ser-202, confirming that the phosphorylation of the Ser-202 site is directly dependent on PP2A activity [52,44]. This also suggests that SET, via the methylation of Leu-309 in PP2Ac, plays an important role in phosphorylation at Ser-202, which is consistently detected in the brains of AD patients. In contrast, we did not observe a negative correlation between the level of methylated PP2A and tau hyperphosphorylation at Ser-422, despite the fact that dephosphorylation at this site depends on PP2A. This suggests that the demethylation of PP2A that is induced by SET does not substantially affect tau phosphorylation at Ser-422. In addition to methylation, PP2A activity can be modulated by other post-translational modifications. We cannot exclude that the interaction of SET with PP2A additionally results in the phosphorylation of PP2A which may be negatively correlated with the phosphorylation of Ser-422. PP2A is phosphorylated at Tyr-307 in PP2Ac by a scr kinase, and this phosphorylated form is also detected in the brains of AD patients [11]. Phosphorylation at this residue or at Ser-41 in the 56α subunit, which is mediated by a PKCα, impairs PP2A activity [53]. In our models, we do not know whether SET induces such post-translational modifications contributing to the impairment of PP2A activity.

PP2A dephosphorylates tau, which leads to the stimulation of several tau kinases [53,54]. In our study, cytoplasmic SET inhibited PP2A; thus, the subsequent tau hyperphosphorylation observed was the result of the absence of tau dephosphorylation. Our data show that Ser-202 becomes highly phosphorylated when cytoplasmic SET inhibits PP2A activity, in contrast with the low phosphorylation at Ser-422 in most studies. However, phosphorylation at Ser-422 is almost undetectable in humans at the adult stage, emphasizing the relevance of a low detectable phosphorylation, as observed in the brains of AD patients [46]. Ser-202 is principally dependent on GSK3-β [44] whereas Ser-422 is a good substrate for MAP kinase but not for GSK3-β [46], suggesting that different sites of tau phosphorylation are regulated by specific kinases. Consequently, cytoplasmic SET, via its inhibition of PP2A would be differently involved in the regulation of specific sites of phosphorylation.

In mouse brain slices, we used OA, which is an exogenous natural inhibitor of phosphatases that inhibits 70% of PP2A activity, as a positive control of tau phosphorylation. In some cases, the pattern of the tau isoforms that were hyperphosphorylated by endogenous cytoplasmic SET or cytoplasmic recombinant SET protein differed from that observed with OA (Figure 4A and Additional file 1: Figure S1), which highlights the physiological relevance of our models. Moreover, both recombinant SET protein and the Jcasp peptide stimulated the hyperphosphorylation of the same tau isoforms (Figure 4A and Additional file 1: Figure S1), which shows that this Jcasp peptide that mimics the unmasked domain of APP after caspase cleavage may be a relevant model for physiopathological investigations of APPcc. In our *ex vivo* model, recombinant SET protein was successfully internalized in the cytoplasm of adult mouse brain slices, where it impaired PP2A methylation and consequently PP2A activity, leading to an accumulation of hyperphosphorylated tau. This model may be used to test molecules that may disrupt the interaction of SET with PP2A and thus prevent the inhibition of PP2A activity [55].

Overall, these data suggest that when SET is in the cytoplasm, it reproduces tau hyperphosphorylation observed in the brains of AD patients by impairing the methylation of PP2A. These findings highly implicate cytoplasmic SET in the progression of the disease.

## Conclusion

We used two *ex vivo* models to show that full-length, cytoplasmic SET, independent of its cleavage, inhibits PP2A activity by decreasing its methylated form, which leads to tau hyperphosphorylation. These findings link the decrease of methylated PP2A (and hence the decrease of PP2A activity) to the translocation of SET to the neuronal cytoplasm, which are two events that are observed in the brains of AD patients. In addition, the negative correlation between the level of methylated PP2A and some, but not all, sites of phosphorylation in tau suggests that tau phosphorylation is regulated by several mechanisms. These data provide an important contribution to the elucidation of the mechanisms involved in AD.

## Methods

### Antibodies

The various antibodies were purchased and diluted as follows.

Anti-Disabled-1, AB5840 (Millipore, Saint Quentin en Yvelines, France) polyclonal rabbit antibody, diluted to 1/1000 for western blotting; anti-I2PP2A/SET (H-120), sc-25564 (Santa Cruz Biotechnology, Heidelberg, Germany) affinity purified rabbit polyclonal IgG for endogenous SET analysis, diluted to 1/2000 for western blotting, to 1/100 for immunocytochemistry, and to 1/100 for SET immunoprecipitation; anti-Histone clone H11-4, MAB 3422 (Millipore) purified mouse monoclonal IgG1, diluted to 1/100 for western blotting; anti-HA mouse monoclonal antibody MMS 101R (Covance, Eurogentecs, Angers, France), for recombinant SET protein analysis, diluted to 1/1000 for western blotting and to 1/200 for immunocytochemistry; anti-APP-Nter anti-Alzheimer Precursor Protein A4 clone 22C11 MAB 348 (Millipore) mouse monoclonal antibody, diluted to 1/1000 for western blotting; anti-Actin, clone C4 MAB 1501R (Millipore) mouse monoclonal antibody, diluted to 1/10000 for western blotting; anti-PP2A C subunit clone 1D6 05–421 (Millipore) mouse monoclonal antibody, diluted to 1/2000 for total protein phosphatase 2A western blotting analysis; anti-methyl-PP2A C subunit clone 2A10 04–1479 (Millipore) mouse monoclonal antibody, diluted to 1/1000 for western blotting; anti-p-Tau (Ser 404) sc-12952 (Santa Cruz Biotechnology) rabbit polyclonal affinity purified antibody, diluted to 1/500 for western blotting; anti-p-Tau (Ser 235) sc-101812 (Santa Cruz Biotechnology) rabbit polyclonal antibody, diluted to 1/500 for western blotting; anti-Tau (pSer356) 54975 (AnaSpec, San José, CA, USA) rabbit polyclonal antibody, diluted to 1/100 for western blotting; anti-Tau (phospho S422) ab4862 (Abcam, Cambridge, UK), rabbit polyclonal antibody diluted to 1/1000 for western blotting; anti-p-Tau (Ser202), anti-Human PHF-TAU clone AT8 BR-03 (Innogenetics, Courtaboeuf, France) mouse monoclonal antibody, diluted to 1/1000 for western blotting; anti-Tau mouse (TAU-5) mAb 577801 (Calbiochem, Merck Chemicals Limited, Nottingam, UK) mouse monoclonal antibody, diluted to 1/1000 for total tau analysis in western blotting.

The secondary HRP-antibodies used for the detection of chemiluminescence were Goat anti-mouse IgG (H + L)-HRP conjugate 170–16516 (Bio-Rad, Marnes-La-Coquette, France), diluted to 1/5000 and ECL anti-rabbit IgG-HRP NA9340V (GE Healthcare, Dutscher, Brumath, France) diluted to 1/2000.

Secondary antibodies used for immunocytochemistry were Donkey anti-rabbit cy3 diluted to 1/500 and Donkey anti-mouse cy3 diluted to 1/200 (both from Jackson Immuno-research, Interchim, Montluçon, France).

### Jcasp, JA peptides and recombinant SET protein

Peptides of APP 649–664 (Jcasp) and Y653A Jcasp (JA) both containing penetratin at their N-termini were chemically synthesized (95-98% purity, Neomps, Strasbourg, France).

Recombinant SET protein was synthesized as previously described [27].

### Ethics statement

All animal procedures were carried out according to French and European Union regulations. The protocols of animal anesthesia were performed according to the recommendations of the European Economic Community (86/609/EEC) and the French National Committee (87/848) of the French government and were approved by the local ethics committee (Direction départementale des services vétérinaires de Paris, service de la protection et santé animales et de la protection de l’environnement).

### Primary cortical neurons

Primary cortical neurons were obtained from E16 Swiss mouse embryos, as described previously [56]. Dissociated cells were plated on polyornithine-coated plastic dishes for biochemical analysis and on glass coverslips for immunocytochemistry at a density of 15 × 10^4^ cells/cm^2^. The Jcasp peptide or its mutant JA peptide (2 μM final concentration) or the recombinant SET protein (4 μM final concentration) were added to the culture medium at 5 days *in vitro* (DIV) and were incubated for 3 h or 5 h 30 min.

### Ex vivo adult mouse brain slices

Male Swiss mice were killed by decapitation and the brains were put on ice. The cerebellum, olfactory bulb, and thalamus were discarded. Cross sections of brain hemispheres were then rapidly cut at 4°C with a McILwain tissue chopper. Slices (300 μm thick) were suspended in 10 ml of modified Krebs-Ringer phosphate medium (KPR) (NaCl 123 mM, KCl 2 mM, CaCl_2_ 2.6 mM, MgSO_4_ 0.67 mM, KH_2_PO_4_ 1.2 mM, Glucose 5.9 mM and NaHCO_3_ 27 mM) that was equilibrated to pH 7.4 with a mixture of O_2_/CO_2_ (95/5) at room temperature for 30 min, as previously described [57]. The slices were then washed 3 times for 10 min each. The slices were finally resuspended in 3 ml of fresh KPR and were incubated in a water bath at 37°C for 15 min under a constant flow of O_2_/CO_2_ (95/5). The slices were then homogeneously distributed into 4 well culture dishes (about 16 wells for one mouse brain per experiment) that were put in a large box placed in a water bath at 37°C under a constant flow of O_2_/CO_2_. OA, Jcasp and JA peptides, and recombinant SET protein were added to wells at final concentration of 1 μM, 2 μM, 2 μM, and 4 μM, respectively and were incubated at 37°C for 2 h (OA) or 5 h 30 min (Jcasp, JA and SET). At the end of incubation the brain slices were distributed in eppendorf tubes, and were rinsed by decantation three times with the KPR medium to eliminate all traces of OA, Jcasp, JA and SET. Slices were then homogenized with a tissue grinder (Kontes glass CO) in different lysis buffers according to the type of analysis. Protein concentration was determined with the Micro BCA Protein Assay Kit (Fischer Scientific, Illkirsch, France).

Slices dedicated to the analysis of tau phosphorylation by western blotting were extracted in Dulbecco’s PBS containing DNaseI (30 μg/ml), 1% Triton X-100, protease inhibitors (Complete, Roche Diagnostics, Mannheim, Germany) and phosphatase inhibitors (2 mM Na_3_VO_4_ and 100 mM NaF).

For the analysis of phosphatase 2A (PP2A) activity after PP2A immunoprecipitation, the same extraction buffer without phosphatase inhibitors was used.

For the analysis of total phosphatase activity, slices were rinsed twice in 50 mM Tris buffer, 0.1 mM CaCl_2_ pH 7.5 containing protease inhibitors and were extracted in the same buffer.

For SET and PP2A co-immunoprecipitation experiments, slices were washed three times in KPR containing protease and phosphatase inhibitors.

### Nuclear and cytoplasmic fractionation

Mouse cortical primary neurons (5 DIV) were incubated with Jcasp peptide (2 μM) and mouse brain slices were incubated with either Jcasp peptide (2 μM) or recombinant human SET protein (4 μM) for 5 h 30 min. Primary neurons and mouse brain slices that were not incubated with any peptide or recombinant protein under the same conditions were used as controls. Cells and tissue slices were rinsed once in cold phosphate buffer saline PBS before being subjected to sub-cellular fractionation. Nuclear and cytoplasmic fractionation was carried out with the Nuclei EZ Prep Nuclei Isolation Kit (Sigma-Aldrich, Saint-Louis, MO, USA) following manufacturer’s instructions. Briefly, scrapped neurons and brain slices were lysed in cold Nuclei EZ lysis buffer containing protease inhibitors. The homogenised extracts were incubated on ice for 5 min and were then centrifuged for 15 min at 500 × g at 4°C. The supernatants were immediately transferred to clean tubes and centrifuged twice for 15 min at 5000 × g. The final clarified supernatant corresponding to the cytoplasmic fractions were stored at -80°C until use for western blotting. In parallel, the pelleted nuclei were washed in 4 ml of ice cold Nuclei EZ lysis buffer and then were resuspended carefully and incubated for 5 min on ice. Washed nuclei were then collected by centrifugation for 15 min at 500 × g at 4°C. Each pellet was resuspended in 100 μl of Nuclei EZ storage buffer and stored at -80°C until use for western blotting.

### Western blotting

Standard SDS-PAGE western blotting was performed with 10% Tris–HCl acrylamide home-made gels or precast 10% gels (Bio-Rad). After electrophoresis, proteins were transferred onto PVDF membranes (Millipore). Briefly, membranes were then blocked in 5% low fat milk in Tris buffer saline, 0.02% Tween 20 (TBST) pH 7.4 and were incubated overnight at 4°C with primary antibodies diluted in TBST. After 5 washings in TBST they were incubated with secondary peroxidase-conjugated antibodies for 1 h at room temperature. After 5 washings, the peroxidase signal was visualized by enhanced chemiluminescence, and if necessary, were enhanced by Super Signal West Femto Maximum Substrate (Fischer Scientific). The ECL films were scanned with a GS-800 Calibrated Densitometer. Image J software was used for the densitometric quantification of protein bands.

For western blotting of nuclear and cytoplasmic fractions, histone was used as nuclear marker and Disabled-1 (Dab1) or APP was used as cytoplasmic marker.

### Immunocytochemistry for SET subcellular localisation

Cells were fixed with 4% paraformaldehyde for 30 min at room temperature, were rinsed 3 times in (PBS) and were saturated for 1 h at 37°C with 10% fetal calf serum in PBS, containing 0.2% Triton X-100. The cells were incubated with antibodies (anti-SET or anti-HA, diluted to 1/200) for 1 h at 37% in saturation buffer, were then rinsed 3 times in PBS, and were incubated with anti-rabbit cy3 or anti-mouse cy3 for 1 h at 37°C. After 3 rinses in PBS the coverslips were mounted in a medium containing DAPI for epifluorescence microscopy. For confocal microscopy, nuclei were labeled by 0.1 μM TO-PRO-3 (Life Technologies, Illkirsch, France) for 15 min at room temperature, the coverslips were then rinsed 3 times in PBS and were mounted with Fluoromount-G (Clinisciences, Nanterre, France).

Immunofluorescence was analyzed by epifluorescence microscopy with an Axioplan 2 Zeiss with a x63 plan Apochromat oil immersion objective (numerical aperture 1.4). Digital images were acquired with a Sony DXC S500 colour digital camera. All pictures were shot at the same exposure time for each experiment.

The cells were also examined with a TCS SP5 confocal imaging system equipped with DPSS 561 nm and HeNe 633 nm lasers (Leica Microsystems, Mannheim, Germany). Eight bit digital images were collected in sequential mode with a x63 plan Apochromat oil immersion objective, a numerical aperture of 1.4, a zoom of 3 and the pinhole size "airy 1".

Microscopy was performed at the PICPEN platform (INSERM, UMR 894).

### Total phosphatase activity assay

The mouse brain slice lysates were centrifuged at 16000 × g for 15 min at 4°C, then NiCl_2_ (2.5 mM final) and p-nitrophenyl phosphate (Sigma-Aldrich) (1 mg/ml final) were added to supernatants, followed by incubation for 30 min at 37°C. The reaction was stopped by the addition of 13% K_2_HPO_4_ and the absorbance of released p-nitrophenol was read at 405 nm.

### PP2A immunoprecipitation and PP2A activity assay

The mouse brain slice lysates were centrifuged at 16000 × g for 15 min at 4°C, and the supernatants were incubated with the PP2A antibody for 2 h (5 μg for 500 μg of protein) at room temperature, followed by incubation with protein G Sepharose (Fisher Scientific) for 1 h at room temperature. The immunoprecipitates were washed carefully twice with 50 mM Tris pH 7.5, 50 mM NaCl followed by two washes in 50 mM pH 7.5 Tris, 0.1 mM CaCl_2_ pH 7.5. The immunoprecipitates were then suspended in 50 mM Tris, pH 7.5 assay buffer containing 0.1 mM CaCl_2_, 2.5 mM NiCl_2_ and 1 mg/ml p-nitrophenyl phosphate as a substrate, and were incubated at 37°C for 30 min. The reaction was stopped by the addition of 13% K_2_HPO_4_ and the absorbance of released p-nitrophenol was read at 405 nm.

### SET-PP2A co-immunoprecipitation

Mouse brain slices incubated for 5 h 30 min with or without recombinant SET protein were prepared as described above. Washed slices were homogenized with a tissue grinder (Kontes glass CO) in immunoprecipitation buffer (Hepes 10 mM, NaCl 150 mM, CaCl_2_ 2 mM, Triton X-100 1% pH 7.4) containing protease and phosphatase inhibitors and the resulting lysates were centrifuged at 16000 × g for 45 min at 4°C. Proteins were quantified with the Micro BCA Protein Assay Kit (Fischer scientific). Lysates containing 1 mg of protein in 400 μl were pre-cleared by incubation with 30 μl of protein A Sepharose CL-4B (Fischer Scientific) for 1 h at 4°C on a rotating wheel. The clear supernatant was incubated overnight at 4°C on a rotating wheel with or without the polyclonal anti-SET. A total of 30 μl of protein A Sepharose CL-4B was then added to each tube for 2 h at 4°C on a rotating wheel. The samples were centrifuged at 1000 × g for 10 min at 4°C, and were then washed twice with immunoprecipitation buffer and a third time in the same buffer without Triton X-100. Samples were then suspended in NuPage LDS Sample Buffer (Bio-Rad) adjusted to 5% β-mercaptoethanol and 5% SDS, were boiled for 5 min and were centrifuged for 5 min at 16000 × g at room temperature. The supernatants were then analyzed by western blotting.

In some experiments, the Pierce Crosslinking Kit (Fischer Scientific) was used according to the manufacturer’s procedure. Briefly, the pre-clearing step was performed with a control agarose resin for 1 h at 4°C on a rotating wheel. The polyclonal SET antibody was covalently coupled to protein A/G Plus agarose and was incubated with the pre-cleared lysate over night at 4°C on a rotating wheel. After four washes - the last without Triton X-100 - the immunoprecipitated proteins were eluted with 30 μl of the kit elution buffer in a tube containing 5 μl of 1 M Tris pH 9.5 to neutralize the low pH of the elution buffer.

### Real time PCR for mRNA quantification

Neurons at 5 DIV were incubated for 1 h 30 min, 2 h 30 min, 3 h 30 min or 5 h 30 min with 2 μM of Jcasp peptide or 2 μM of Penetratin. The medium was removed and the cells were washed once in PBS and then collected by scraping. Total RNA was extracted with the RNeasy mini kit and was treated with DNaseI (Qiagen, Courtaboeuf, France). cDNA was prepared from total RNA samples with the High Capacity cDNA Reverse Transcription kit (Life Technologies, Saint Aubin, France). Briefly, 20 μl of reaction mixture containing 100 ng of total RNA, 1X reverse transcription buffer, 1 mM of each dNTP, 1X reverse transcription random primer and 50U of MultiScribe reverse transcriptase were incubated at 25°C for 10 min followed by incubation at 37°C for 2 h. The reaction was stopped by heating at 85°C for 5 sec. Real time PCR was performed with an Abi Prism 7000 and the Absolute SYBR Green ROX Mix (Fischer Scientific). A total of 25 ng of cDNA was mixed with 0.07 mM of each primer, 1X SYBR Green Mix and PCR-grade water to a final volume of 25 μl. The cycling conditions for all primers (*Set* forward: TTACTGACCATTCTGACGCA, *Set* reverse: CTGCCTCTCCTTCTTCATCA, *Gapdh* forward: CCAACACTGAGCATCTCCCT and *Gapdh* reverse: GGGTGCAGCGAACTTTATTG) were 50°C for 2 min, then 95°C for 15 min followed by 40 cycles consisting of two steps, 15 sec at 95°C and 1 min at 60°C. The PCR program was completed by a melting temperature analysis consisting of 15 sec at 95°C, 20 sec at 60°C and then steps through which temperature ranged from 60 to 95°C. Amplification plots were produced to calculate the threshold cycle (Ct) and standard curves of Ct versus log cDNA dilution were generated for both target (SET) and reference (GAPDH) genes. All reactions were done in triplicate and the average Ct was used. Relative quantification was calculated with the 2^-ΔΔCt^ method. Gene expression in Jcasp peptide treated neurons was calculated relative to that of Penetratin treated cells.

### Statistical analysis

Significance was assessed with the analysis of variance (ANOVA) and post-hoc Scheffe’s test (StatView). Results were expressed as mean value ± SEM.

## Competing interests

The authors declare that they have no competing interests.

## Authors’ contributions

SC, CC, BA conceived and designed experiments. SC and CC performed biochemical experiments. LH and LG performed immunocytochemical experiments. SC, CC, CR and BA analyzed data. SC, CC, BA wrote the paper. All authors read and approved the final manuscript.

## Supplementary Material

Additional file 1: Figure S1 **Differences in hyperphosphorylated tau isoforms in cells treated with OA, Jcasp peptide or recombinant SET protein.** Mouse brain slices were treated with recombinant SET protein or Jcasp peptide for 5 h 30 min, and proteins were then extracted and prepared (40 μg per lane) for western blotting. Various residues that may be hyperphosphorylated on tau were analyzed. Okadaic acid (OA) was used as positive control. Brain slices not treated with recombinant SET were used as a control (Ctrl). Jcasp and SET induce hyperphosphorylation of the same tau isoforms. In some cases these hyperphosphorylated tau isoforms differ from those induced by okadaic acid (OA). Representative examples of two independent experiments are shown **(A, B)**. Note that for the same mouse brain, the tau isoforms phosphorylated at the Ser-422 epitope were identical for OA and recombinant SET, contrary to what we observed for Ser-202 **(B)**. Click here for file
