# Supplementary figures and images for: Cytoplasmic SET induces tau hyperphosphorylation through a decrease of methylated phosphatase 2A
Source: BMC Neurosci. 2014 Jun 30;15:82. doi: 10.1186/1471-2202-15-82 (PMC4086270; doi:10.1186/1471-2202-15-82)

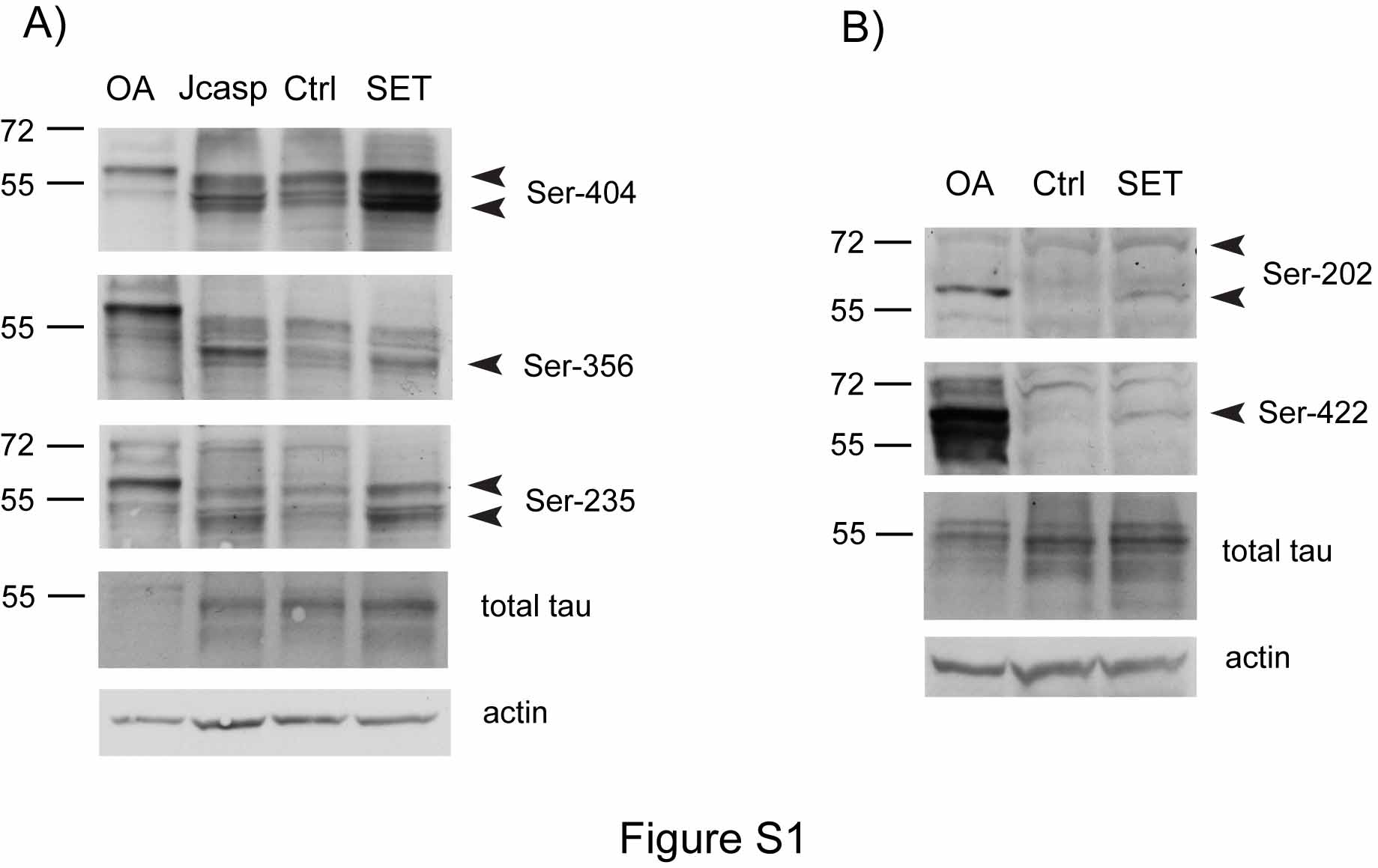

Supplement: Additional file 1: Figure S1 — Differences in hyperphosphorylated tau isoforms in cells treated with OA, Jcasp peptide or recombinant SET protein. Mouse brain slices were treated with recombinant SET protein or Jcasp peptide for 5 h 30 min, and proteins were then extracted and prepared (40 μg per lane) for western blotting. Various residues that may be hyperphosphorylated on tau were analyzed. Okadaic acid (OA) was used as positive control. Brain slices not treated with recombinant SET were used as a control (Ctrl). Jcasp and SET induce hyperphosphorylation of the same tau isoforms. In some cases these hyperphosphorylated tau isoforms differ from those induced by okadaic acid (OA). Representative examples of two independent experiments are shown (A, B). Note that for the same mouse brain, the tau isoforms phosphorylated at the Ser-422 epitope were identical for OA and recombinant SET, contrary to what we observed for Ser-202 (B). [file 1471-2202-15-82-S1.jpeg]
